# Parents’ Motivations for Calling an Out-of-Hours Helpline: Qualitative Study

**DOI:** 10.2196/66780

**Published:** 2025-05-15

**Authors:** Liv Borch-Johnsen, Fredrik Folke, Marianne Sjølin Frederiksen, Morten Schrøder, Gorm Greisen, Stine Lund, Vibeke Zoffmann, Caroline Gren, Tine Tjørnhøj-Thomsen, Dina Cortes

**Affiliations:** 1 Department of Pediatrics and Adolescent Medicine Copenhagen University Hospital – Amager and Hvidovre Copenhagen Denmark; 2 Emergency Medical Services Capital Region Copenhagen Denmark; 3 Department of Clinical Medicine University of Copenhagen Copenhagen Denmark; 4 Department of Cardiology Copenhagen University Hospital – Herlev and Gentofte Copenhagen Denmark; 5 Department of Pediatrics and Adolescent Medicine Copenhagen University Hospital – Herlev and Gentofte Herlev Denmark; 6 Department of Pediatrics and Adolescent Medicine Copenhagen University Hospital – Rigshospitalet Copenhagen Denmark; 7 Department of Neonatology Copenhagen University Hospital – Rigshospitalet Copenhagen Denmark; 8 Department of Pediatrics and Adolescent Medicine Copenhagen University Hospital North Zealand Hilleroed Denmark; 9 The Interdisciplinary Research Unit of Women’s, Children’s and Families’ Health Copenhagen University Hospital – Rigshospitalet Copenhagen Denmark; 10 Department of Public Health University of Copenhagen Copenhagen Denmark; 11 Centre for Quality and Patient Safety Research in the Institute for Health Transformation School of Nursing and Midwifery Deakin University Geelong Australia; 12 Department of Pediatrics and Adolescent Medicine Helsingborg Hospital Helsingborg Sweden; 13 National Institute of Public Health University of Southern Denmark Copenhagen Denmark

**Keywords:** pediatrics, telehealth, mHealth, qualitative research, acute disease, child health, digital intervention, primary care knowledge, children, young, medical helpline, MH1813, interviews, parental motivations, medical services

## Abstract

**Background:**

Young children often get sick, and although they usually do not need treatment, it can be distressing for parents and lead to a high rate of urgent health care use. As the demand for out-of-hours services grows, understanding parents’ concerns and needs when caring for an ill child is crucial for designing interventions that support informed health-seeking decisions.

**Objective:**

This study aimed to investigate why parents contacted a Medical Helpline, their expectations regarding the call, and how their situation changed following telephone triage.

**Methods:**

Parents who contacted an out-of-hours Medical Helpline in Denmark participated in semistructured interviews that were analyzed using Braun and Clarke’s 6-step approach to thematic analysis.

**Results:**

A total of 39 interviews were conducted. Our analysis led to three key themes: (1) parental uncertainty in decision-making: caring for an ill child was associated with stress and uncertainty. Parents lacked the tools to differentiate between acceptable symptoms and signs of severe illness, resulting in catastrophic thinking; (2) validation: parents contacted the medical helpline to validate their assessment and share responsibility with a health care professional; they experienced a conflict between responsible health care usage and the need for reassurance; and (3) feeling safe at home: when the health care professional demonstrated competence, recognized parents’ emotions, and dedicated time to explain the symptoms, parents felt empowered to manage their child at home through telephone consultation.

**Conclusions:**

Uncertainty in assessing a sick child’s symptoms can lead parents to seek reassurance and validation by contacting a medical helpline. Telephone consultations often enable parents to manage their children at home. Interventions that help parents distinguish between mild and severe symptoms, and accept frequent illnesses as a normal part of childhood, could reduce stress and reliance on health care services.

## Introduction

Young children often fall ill, leading to more out-of-hours health care usage compared with other patient age groups [1-3]. Fortunately, severe illness in children is rare [4]. In a study of 16,000 children under 5 years admitted to a pediatric department with fever, only 7% had a severe infection [5]. The high rate of health care usage is often driven by a fear of making the wrong decisions and failing to recognize severe symptoms, particularly in young children [5-10]. The presence of fever, being first-time parents, and a lack of social support are associated with an increased need for health care services [8,11,12].

In the Capital Region of Denmark, the Medical Helpline 1813 (MH1813), a free out-of-hours service, receives over 250,000 calls each year regarding children, with 40% of those calls leading to a physical evaluation at the hospital [13]. Serving 1.9 million citizens, MH1813 uses health care professionals who triage acute, non–life-threatening illnesses and injuries across the region’s 11 hospitals [11]. Research indicates that some calls to medical helplines stem from the convenience of immediate access rather than medical urgency, resulting in inefficient use of health care resources [10,12].

Empowering parents to care for sick children at home without requiring medical intervention could ease parental worries and lessen the demand for urgent medical consultations [14-21]. Many parents look for web-based health information [22,23], but there is a lack of reliable, high-quality resources to reassure them during the stressful experience of caring for a sick child.

In response to these challenges, we developed “Tips from Pediatricians,” a series of 9 short video tutorials on common symptoms in acutely ill children [21]. We evaluated this intervention in a randomized controlled trial (RCT), in which parents calling MH1813 received video tutorials instead of telephone triage [20]. This qualitative study explores the help-seeking behaviors of parents facing acute childhood illnesses, providing context to understand the motivations behind using a medical helpline, an essential complement to insights gained from the RCT. As the demand for out-of-hours services grows, understanding the needs of parents caring for an ill child is crucial for designing effective interventions that support parents in making informed and appropriate health-seeking decisions.

## Methods

### Aim and Study Design

This study aimed to investigate why parents contacted a Medical Helpline, their expectations regarding the call, and how their situation changed following telephone triage. We chose a qualitative design to obtain data on the parents’ perspectives on these key aspects. The study is based on semistructured interviews with parents who called MH1813 between June and December 2021.

### Setting, Recruitment, and Participants

The setting was MH1813, a free out-of-hours medical Helpline serving the Capital Region, Denmark. Approximately 300 parents were invited from the call database at MH1813 via text message 3 days following their MH1813 call. We used a convenience sampling strategy [24], contacting only those who expressed interest in participating. When contacting the parents, we had no information other than the child’s age. Of the 60 parents who expressed interest, 21 were unreachable at the scheduled time.

The invitation included prompts to encourage reflection before the interview, such as, “When I think back on the situation when I contacted MH1813, the first thing that comes to mind is…” (Multimedia Appendix 1). These prompts were related to the research questions and aimed to foster deeper, more meaningful insights during the interview. The 3-day delay was chosen to reduce additional stress associated with caring for an ill child and to minimize social desirability bias by alleviating parents’ concerns that the interviewer might influence their child’s treatment. This was to decrease the likelihood of parents withholding criticism during the interview. The median time from the initial call to the interview was 6 days [25].

**Figure 1:**
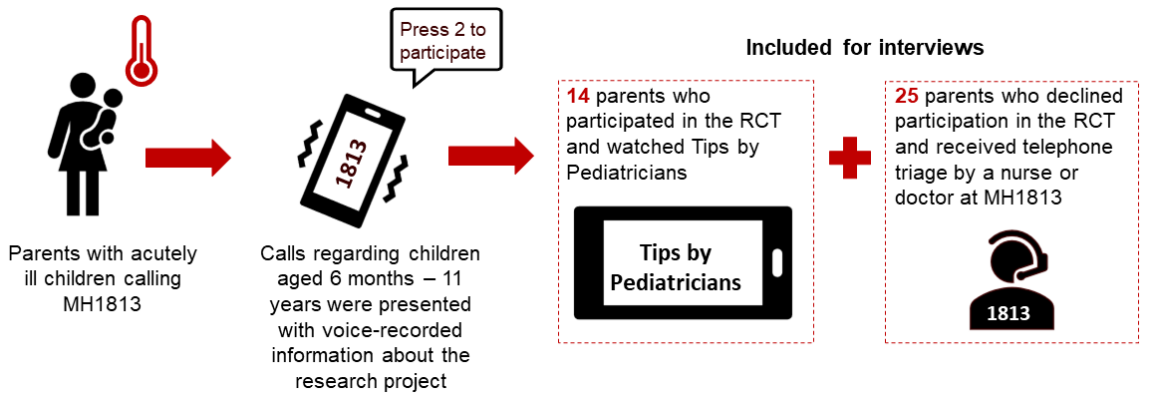
Included parents for interviews. MH1813: Medical Helpline 1813; RCT: randomized controlled trial.

The inclusion criteria were Danish-speaking parents calling MH1813 regarding children aged 6 months to 11 years with acute illnesses. We included 14 parents who had participated in the RCT and watched video tutorials, along with 25 who declined the RCT but received telephone triage from a nurse or a doctor (Figure 1) [20,21]. The recruitment process was designed to address potential differences between RCT participants and nonparticipants, as we also aimed to examine barriers to participation in the RCT and participants’ experiences with the videos. These findings will be reported separately.

Parents unable to participate in a telephone interview or calling regarding an injury were asked to self-exclude, as the videos did not cover injuries, and they may have differed in their motivations for calling MH1813. A total of 6 interviews appeared to be related to injuries of the child. Despite our initial exclusion criteria, we chose to include these interviews out of respect for the parents, who dedicated their time and provided valuable and unexpected insights.

### Interviews

The first author (LB) conducted semistructured interviews, which lasted between 9 and 23 minutes [26,27]. The interview guide, developed by the research group, included open-ended questions regarding the parents’ motivations for calling MH1813 (Multimedia Appendix 1). Telephone interviews were scheduled at the parents’ convenience, with a priority on prompt timing to minimize recall bias [28]. They were recorded with parents’ consent and transcribed verbatim by LB and CG [25,29]. Coauthor VZ, a senior qualitative researcher, reviewed the first 2 interviews to assess parents’ responses and ensure they aligned with the research objectives. She provided feedback on interview techniques, which resulted in minor adjustments to the interview guide.

We collected data on parental and child characteristics (number and age of children in the household, main symptom of the child, parental gender, age, occupation, and education), which can be found in Table S1 in Multimedia Appendix 1. Furthermore, all parents mentioned unprompted whether the child had been referred to the hospital during their MH1813 call (Table S1 in Multimedia Appendix 1). Data saturation was achieved when we observed recurring themes and responses emerging from the data, while also maintaining a diverse representation of gender, age, occupation, and education among the parents [24].

### Data Analysis

Thematic analysis followed Braun and Clarke’s 6-step approach to identify patterns and themes in the data [30,31]. Data familiarization involved reading and rereading, followed by inductive coding using the computer software package NVivo14 (Lumivero). LB and CG coded the transcriptions. For example, when parents mentioned “age,” “high fever,” and “breathing” as concerns during a child’s illness, these were grouped under the subtheme “worries during the child’s illness.” Overall, 2 study team members with expertise in qualitative methods supported this process (VZ and TTT).

### Team and Reflexivity

The principal author, LB, a medical doctor with experience assisting parents seeking urgent care, conducted all the interviews. Most of the research team had a combined professional experience in managing ill children, as well as personal experience, which may enhance their empathy and comprehension of parents’ situations. While we initially expected that parents called MH1813 primarily out of a wish for referral to a hospital, the interviews revealed a more complex set of motivations. To mitigate bias, the research team discussed emerging themes to ensure a balanced interpretation. Besides LB, the team included 6 pediatricians (MSF, MS, GG, SL, CG, and DC), covering general pediatrics, neonatology, acute pediatrics, and nephrology, as well as a cardiologist (FF) specializing in prehospital research, a nurse (VZ) and an anthropologist (TTT), both with extensive experience in qualitative research and a diverse nonmedical perspective on parents’ motives.

### Ethical Considerations

The study was registered and approved by the Center for Regional Development (P-2020-975, P-2021-799, and R-21052012), adhering to the Danish Code of Conduct for Research Integrity [32] and the guidelines of the Danish Data Protection Agency. Participation was voluntary, with the option to withdraw at any time, and all parents provided either oral or written consent in accordance with the Declaration of Helsinki [33]. Calling a medical helpline can be sensitive, raising concerns about privacy and data security. We anonymized all data, used secure platforms for interview recordings and transcriptions, and ensured strict confidentiality. Interviews were conducted at the parents’ convenience. Acknowledging that the topic might be sensitive for some parents, we allowed them to guide the conversation and express their thoughts while still covering the questions in the interview guide, making them feel heard instead of letting the guide dictate the direction of the conversation. All parents received contact information for LB in case any questions arose after the interview; however, no parents reached out. Patients or the public were not involved in the design or conduct of the study. The Standards for Reporting Qualitative Research were adhered to when preparing this paper (Multimedia Appendix 2) [34].

## Results

### Overview

The demographic and professional characteristics of the 39 participants are described in Table 1. The median age of the interviewed parents was 38 (IQR 33-43) years, with twice as many females (26/39, 67%) as males (13/39, 33%). Most parents had a bachelor’s degree or higher (25/39, 64%).

**Table 1:** Demographic and professional characteristics of the participants.

Characteristics		Total (N=39)
Age of the parent calling years, median (IQR)		38 (33-43)
**Sex, n (%)**		
	Male	13 (33)
	Female	26 (67)
**Level of education, n (%)**		
	Lower secondary education (ISCED 2011^a^ level 0-3)	2 (5)
	Upper secondary education (ISCED 2011^a^ level 4-5)	12 (31)
	Bachelor’s degree or higher (ISCED 2011^a^ level 6-8)	25 (64)
**Number of children, n (%)**		
	1	18 (46)
	2	17 (44)
	3 or more	4 (10)
Age of the children (years), median (IQR)		4 (2-9)
**The main symptom of the child, n (%)**		
	Fever	19 (49)
	Respiratory symptoms	5 (13)
	Gastrointestinal symptoms	4 (10)
	Injuries	6 (15)
	Other	5 (13)
Number of children referred to hospital for treatment, n (%)		14 (36)

^b^ISCED 2011: International Standard Classification of Education 2011.

A total of 3 key themes were produced from our data. The first describes the circumstances preceding the call to MH1813, encapsulating the feelings parents experience when caring for an ill child and the actions leading up to the call to MH1813: “Parental uncertainty in decision-making.” The following 2 themes describe the motivations prompting the call and the impact of the telephone triage: “Validation” and “Feeling safe at home.”

### Parental Uncertainty in Decision-Making

When asked about the circumstances leading up to calling MH1813, parents unanimously described caring for an ill child as challenging and highly stressful, primarily due to uncertainty in assessing the child and whether the symptoms were signs of severe disease. These feelings were intensified by the emotional responsibility parents feel when their child is sick, compared with if it was the parent who was ill themself.

Watching your child fall ill triggered a unique and unmatched worry and created an intense need to ensure their safety (quote Q1).

Q1: *The worry you feel when there's something wrong with your children is unlike any other concern in life, at least in my world.* [Parent 23]

Parents struggled to assess symptoms, especially in younger children, where parents had to rely on subtle behavioral cues, increasing their responsibility as the child’s advocate. Children were seen as fragile; their condition could deteriorate more quickly. The inability to communicate increased the risk of missing critical warning signs, leading parents to worry and constantly monitor their children. Several parents believed that children should be given a higher priority in the health care system (Q2 and Q3).

Q2: *There should be a separate way of handling children. I can assess how I feel and take care of myself. A child is entirely dependent. There are things we can’t be told, like where it hurts. We must check constantly. It’s like having a pet; you are responsible for ensuring it’s okay because it can’t speak up, act, or care for itself. And that’s precisely how it is with children, too.* [Parent 38]

Q3: *I think one should be extra attentive to young children. It may just be because I am the father. But it can develop into something serious very quickly. And you are very worried about such a small child. And the child cannot tell what is wrong.* [Parent 36]

Parents felt torn between waiting to see if symptoms improved on their own and seeking immediate medical advice. Rather than focusing on positive indicators (eg, eating, drinking, and alertness), parents fixated on signs of severe disease and whether they could manage alone or needed professional advice (reflecting a tension between rational decision-making and emotional uncertainty). This was exacerbated by catastrophic thoughts about breathing arrest or febrile seizures (Q4).

Q4: *I knew she had a virus, probably just that RS virus. I was just afraid that it was severe, that she would suddenly stop breathing. When you sit there, especially as a first-time parent—I hope it gets better with the second one— you must figure out if this is urgent, if you need to call (the doctor).* [Parent 8]

Several parents felt uncertain due to the lack of a precise diagnosis, which led to a sense of losing control (Q5). Conditions such as high fever, breathing difficulties, pain, or persistent symptoms increased the fear of severe illness. However, parents’ perceptions of these symptoms varied significantly. Nighttime triggered anxiety as parents felt ill-equipped to assess a tired child and, consequently, could not rule out severe illness. Mothers and fathers did not differ meaningfully in what made them worry, but a few fathers highlighted that it was typically the mother who took charge when the child went ill.

Q5: *I know that 1000 different things can cause fever. Luckily, 950 of them are harmless. But when I am uncertain about the doctor’s advice, I know that no one has been wrong so far. I want control. To know 100% what is happening with your child.* [Parent 39]

Many parents appeared well-informed about managing common childhood illnesses and had already tried home remedies or conducted web-based research before calling MH1813. Parents often experienced information as contradictory or worried about its quality and trustworthiness. They described a preference for government-authorized health sites. However, their web-based search behavior led them to the top results of search engines rather than consulting these trusted sources directly. A few refrained from internet searches, feeling unqualified to interpret medical information (Q6).

Q6: *When it comes to medicine and being able to piece things together and make a diagnosis… I don’t think I have the necessary background... I am an engineer, so if I read something on Google, I might diagnose myself and conclude that whatever I have will kill me.* [Parent 23]

Many parents had also contacted relatives with medical backgrounds, and grandmothers were referred to as “encyclopedias of childhood diseases.”

Overall, the parents’ decision-making focused on assessing the severity of their child’s illness rather than monitoring gradual improvements. Their experience reflected being torn between managing uncertainty, containing their anxiety, and initiating medical contact. Being a good parent was strongly associated with seeking timely medical evaluation, driven by a pronounced fear of waiting too long and possibly missing signs of severe disease and delaying care.

### Validation

The call to MH1813 served as validation that what the parent was doing was correct and as emotional support by shifting responsibility to health care professionals. Many parents faced the dilemma of seeking reassurance without overburdening the health care system. Most parents called to stay ahead of the illness and avoid hospital referrals. Parents with a health care background sought validation just like those without it.

For most parents, the purpose of the call was to consult a health professional for reassurance that what they were doing was correct and to share the responsibility of assessing their child (Q7).

Q7: *Because this assessment (of the child)… It often lies with us parents…. I think that what you are most afraid of (as a parent) is to misjudge your child.*

MH1813 provided both medical advice and emotional support to parents. Although worst-case scenarios lingered in their minds, parents recognized these situations were unlikely to occur. During the interviews, they described themselves as not overly worried when calling MH1813. They sought to rule out severe conditions, describing themselves as overcautious (Q8). However, it is essential to note that the interview was conducted after the symptoms had subsided, whereas the real-time decision-making to call MH1813 showed genuine worry.

Q8: *And the thing about wondering should I/should I not (call MH1813) … What should I do now to be a competent parent? I needed guidance to navigate whether it was okay that he had a high fever. I wasn’t worried because we could wake him up, and he didn’t have retractions. It was to be one step ahead.* [Parent 32]

Parents expressed a lack of confidence in assessing their child, calling MH1813 to understand whether their child’s symptoms were within the acceptable range during the illness. Calling to consult symptoms with health professionals was also seen as a part of competent parental caregiving (Q8). Others also called MH1813 out of fear of failing to act in time (Q9).

Q9: *I was afraid of missing something, that he’d get worse and worse overnight, and that we’d then be told, once we brought him in, that we should have reacted sooner.* [Parent 29]

Some parents found that their child had a unique representation of symptoms when sick, such as a higher pain tolerance than other children, which, in their view, complicated assessment and increased the risk of missing severe symptoms, necessitating a second opinion by a health professional.

The parents seemed aware of not overburdening the health care system, potentially diverting resources from more seriously ill children, and felt conflicted about calling MH1813. Others believed they were taking a more cost-conscious approach by calling MH1813 early during their child’s illness to manage symptoms and avoid hospitalization (Q10). At MH1813, the call handler triages the child for home care or hospital assessment. However, most parents called to prevent a referral to the hospital, reaching out to be “one step ahead,” ensuring they did not overlook any warning signs of worsening symptoms or believing that an early phone consultation could help reverse the symptoms.

Q10: *The way we use MH1813… We’ve actually used it a few times... I have not been an extra (time) at the hospital because I have had a 5-minute conversation with someone at MH1813. Everybody wins.* [Parent 37]

Parents’ reasons for not wanting to go to the hospital included anticipated waiting hours and exposure to infections.

Those who had been in contact with their general practitioner before calling MH1813 had been instructed to make contact if symptoms persisted or worsened as a part of a common safety-netting strategy. However, they did not feel equipped to assess whether this improvement was worsening. Many parents had been advised by their general practitioner that it is better to call “one time too many” (Q11).

Q11: *I call as soon as my child gets sick. That’s also what the doctors have told me. They’ve said that if we don’t know what’s wrong with the child, or if the child is crying, it’s better to call than not call.* [Parent 20]

Several parents who called about an injury were primarily seeking a specific outcome. One father, for instance, called to request an x-ray of his child’s ankle to rule out fractures and ensure that his child could participate in a soccer match the following day.

Three parents with health care backgrounds shared how their knowledge sometimes led to unnecessary worry and potential overuse of health care services (Q12).

Q12: *I have a health education. When it’s my child, it’s completely canceled. It’s like she had meningitis or something. I don’t know why it must be like that. That’s just the way it is.* [Parent 37]

Their increased health knowledge seemed difficult to mobilize when caring for their child, requiring the same need to consult MH1813 to validate the assessment as parents without. One parent, a nurse, mentioned that nurses tended to downplay their children’s symptoms, necessitating consults with MH1813, even for mild symptoms. These parents emphasized the importance of external guidance to ensure that their child received the same care as others, steering clear of the role of a health care professional for themselves and their relatives.

### Feeling Safe at Home

The telephone consultation at MH1813 provided a valuable dialogue between the parent and a medical professional, which reduced concern and helped the parent to feel safe and competent in caring for a sick child at home by sharing the responsibility of caring for the child. A “good call” with MH1813 was characterized by ensuring that the parent felt heard and not rushed, which led to empowerment in handling the child at home after the call.

Parents likened MH1813 to having a physical consultation with the general practitioner. However, they found a physical assessment more reassuring than telephone triage. They appreciated when the health professional at MH1813 used an option with video triage, allowing them to visually assess the child through the parent’s smartphone (Q13).

Q13: *I thought that the doctor's solution using video triage was quite nice. Now, I wasn’t insecure about communicating how he [the child] was feeling… He (the doctor), having seen the child with his own eyes, made me quite confident. That I wasn’t a filter between how (name of the child) looked and what I said.* [Parent 32]

“Good calls” were characterized by the call handler taking the necessary time, appearing professional and competent, and emphasizing the role of speaking with an “expert” in the situation (Q14).

Q14: *It’s just the overall user experience. There is something about getting someone on* *the phone who listens, who seems competent, and takes the time that I believe it should take.* [Parent 32]

The call should not feel rushed. The call handler should acknowledge parents’ emotions and reasons for calling MH1813 and take the time to inform them about symptoms and what falls within the “normal range” using nonmedical language (Q15 and Q16).

Q15: *She communicated clearly about what we should observe and assured us that we were doing the right thing. I had no doubt for a second that I was in safe hands. I felt more secure after the conversation. She said she would have felt the same way… It meant a lot that you felt acknowledged.* [Parent 18]

Q16: *He reassured me by confirming my doctor’s advice, telling me what to look out for and when to call again. He provided us with guidelines on what to monitor throughout the night and the following day. Even though it seemed severe, it was still within the normal range, and there wouldn’t be much they could do at the hospital, so we felt comfortable having her at home.”* [Parent 27]

On the contrary, when parents felt unheard by call handlers, they were not comforted by the call but instead left with the impression that their child had to be very sick to be taken seriously (Q17).

Q17: *The child was feeling bad... I wanted to ensure he was okay and didn’t suffer from anything serious. However, the doctor thought it was just a cold. But if a child with a cold is lethargic or sleeps 80% of the time… It should give a reason for concern, right?... It felt like the shit must hit the fan to be taken seriously.* [Parent 36]

The telephone consultation reassured many parents to continue monitoring their child at home after speaking to a health professional, feeling no need for a physical evaluation at the hospital. However, when their worries were dismissed, their sense of vulnerability in managing their child’s care was heightened.

## Discussion

### Principal Findings

This qualitative study investigated parents’ motivations for using out-of-hours services when dealing with acute illnesses in children by exploring why parents contacted a Medical Helpline, their expectations for the call, and how their situation changed following the telephone triage. The data revealed 3 key themes: “Parental uncertainty in decision-making,” the call served as a “Validation,” and it enabled parents to “Feel safe at home” while caring for their child.

Consistent with previous studies [7,8,10,35], parents experienced dealing with sick children as stressful, with uncertainty playing a central role. While demonstrating a high level of health knowledge, such as recognizing general behavior, fast breathing, high fever, and dehydration, they struggled to define an acceptable range. In line with other studies, the primary concern was to rule out severe disease, leading to catastrophic thinking [36]. Parents could rationally acknowledge these “worst case scenarios” were highly unlikely but could not dismiss these thoughts before consulting MH1813, even if the child had been consulted by the general practitioner hours before. The need for reassurance was increased by the emotional responsibility to care for a sick child, even for those with health care backgrounds themselves.

The purpose of calling MH1813 was mostly to alleviate the uncertainty, validate the parental assessment, and be guided on signs of severe disease to keep caring for the child at home. Parents were often not highly worried when calling MH1813, and few sought hospital referrals. About one-third of the children were referred to the hospital for physical evaluation (Table 1). This aligns with MH1813 call patterns; previous studies have shown that many children are sent home with home care advice [20,23]. We found that the dialogue between the parent and a medical professional reduced concern and helped the parent to feel safe and competent in caring for a sick child at home by sharing the responsibility of caring for the child. This interaction not only alleviates parental concerns but also empowers them with the necessary knowledge and confidence to manage their child’s illness.

Parents described an intense inner conflict about calling MH1813. While they did not want to take up health care resources unnecessarily, they also felt a strong responsibility to be cautious, act preventively, and ensure timely treatment of their child. Before calling MH1813, many tried to find answers in other ways, such as through family members and web-based health information. Parents voiced apprehensions about the quality of web-based health information and relied on Google’s top results, underscoring the significance of search engines in shaping health-seeking behavior. One parent highlighted the challenge of interpreting health information without health education, aligning with theories that parents often struggle to trust their instincts across various aspects of childcare [37,38]. In Denmark, the National health authorities encourage parents to follow structured guidance on childcare. While these initiatives are well-intentioned, they may decrease parental ability to trust their own assessment. In addition, private companies capitalize on parental concerns by offering courses and products emphasizing potential dangers and risk avoidance, contributing to a culture of “paranoid parenting” or “risk aversion [37,38].”

This study builds on previous research illustrating parents’ challenges in managing childhood illness. However, this study also highlights a critical gap in health care communication, particularly in safety netting. Most health care information emphasizes warning signs while failing to explain what falls within an acceptable range. While caring for an ill child is inherently stressful, frequent illness is also a natural part of immune system development [39]. Interventions to support parents should help alleviate anxiety. Health information should emphasize the importance of overall general condition over the degree of fever, guide parents in assessing a tired child, normalize prolonged and non-linear recovery (such as lingering coughs after a cold), focus on gradual healing, and reinforce that watchful waiting is an active, responsible decision. Many parents in this study had consulted a general practitioner the same day, and previous studies indicate some parents called MH1813 multiple times within a few days [20]. Such information could provide a more sustainable reassurance, enhance parental empowerment, and potentially reduce health care usage.

### Strengths and Limitations

This study has several limitations. One limitation is that we needed to invite many parents to achieve the 39 interviews. Due to the lack of detailed information on nonparticipants beyond phone numbers and children’s ages, we cannot assess potential biases between those who agreed to be interviewed and those who did not. In addition, many parents in our sample were well-educated, which indicates a more resourceful group. They may differ in how they navigate health care and articulate their concerns compared with parents with different levels of education, which limits generalizability. Furthermore, parents with negative experiences might be less likely to participate.

Twice as many interviewed parents were female, mirroring a Swedish study that found mothers made over two-thirds of calls regarding children to a medical helpline [40]. Fathers expressed similar concerns but noted that mothers usually handled treatment, emphasizing traditional gender roles in seeking assistance. Future research should investigate paternal perspectives to ensure the development of inclusive interventions and communication strategies that recognize diverse caregiving dynamics and promote greater paternal involvement.

The interviews were performed over the phone, which allowed for anonymity and flexibility, making participation easier compared with having to attend face-to-face interviews; however, creating a space where parents felt comfortable to confide in the interviewer may have been more challenging [25,29]. There is also a potential for distraction, such as childcare or commuting, which may have led to shorter responses and less depth. In addition, nonverbal cues were lost, though these can be difficult to interpret reliably [25,29].

Furthermore, the study overlooks how language barriers or cultural heritage may influence helpline use by only including Danish-speaking parents. In Denmark, 1 in 6 people are immigrants or their descendants. While the exact number of non-Danish speakers is unknown, research has consistently shown that cultural and language barriers contribute to health care inequalities.

### Conclusions

This qualitative study identified that parental stress when caring for an ill child was rooted in uncertainties as parents lacked tools to define acceptable symptoms during disease, making it difficult to rule out signs of severe illness. This drove parents to contact a medical helpline for reassurance, validation, and shared responsibility. However, many parents experienced a dilemma between using health care responsibly and seeking reassurance. Telephone consultations with health professionals often empowered parents to manage their children at home. Providing high-quality health care information with concrete advice on the assessment of an ill child, focusing on alleviating concerns and empowering parents, could potentially reduce parental stress and reliance on health care services.

## Appendix

Multimedia Appendix 1 Additional data.

Multimedia Appendix 2 Standard for Reporting Qualitative Research (SRQR).

## Abbreviations

MH1813: Medical Helpline 1813
RCT: randomized controlled trial
