# Supplementary material for: Parents’ Motivations for Calling an Out-of-Hours Helpline: Qualitative Study
Source: J Med Internet Res. 2025 May 15;27:e66780. doi: 10.2196/66780 (PMC12123230; doi:10.2196/66780)
Supplement: Multimedia Appendix 1 [file jmir_v27i1e66780_app1.docx]

**Additional Data**

**Table 1.** Characteristics of the interviewed parents and their children

| **No.** | **RCT participant** | **Age, yrs** | **Gender** | **No. of child-ren within family** | **Ages of children, yrs** | **Educational level (ISCED)** | **Employment status** | **Interview duration, min** | **Time from primary MH1813 call to interview, days** | **Main symptom of the child when calling MH1813** | **Referred to hospital during MH1813 consultation** |
| --- | --- | --- | --- | --- | --- | --- | --- | --- | --- | --- | --- |
| 1 | Yes | 42 | F | 2 | 2,7 | Higher | Fulltime | 11 | 6 | Fever | No |
| 2 | Yes | 29 | M | 1 | 2 | Secondary | Fulltime | 9 | 9 | Fever | No |
| 3 | Yes | 49 | M | 2 | 11,14 | Higher | Fulltime | 21 | 6 | Injury | Yes |
| 4 | Yes | 26 | F | 1 | 2 | Higher | Fulltime | 14 | 5 | Injury | No |
| 5 | Yes | 36 | F | 5 | 3,5,6,12,16 | Higher | Early retirement | 14 | 9 | Diarrhea/vomiting | No |
| 6 | Yes | 38 | M | 2 | 0,3 | Higher | Early retirement | 16 | 8 | Fever | Yes |
| 7 | Yes | 34 | F | 2 | 2,5 | Higher | Fulltime | 19 | 6 | Diarrhea/vomiting | No |
| 8 | Yes | 34 | F | 1 | 1 | Higher | Fulltime | 14 | 9 | Respiratory symptoms | Yes |
| 9 | Yes | 39 | M | 1 | 2 | Higher | Fulltime | 9 | 5 | Fever | No |
| 10 | Yes | 31 | F | 1 | 2 | Higher | Fulltime | 17 | 6 | Fever | No |
| 11 | Yes | 38 | F | 2 | 5,9 | Higher | Fulltime | 12 | 9 | Fever | Yes |
| 12 | Yes | 41 | F | 2 | 10,13 | Higher | Fulltime | 17 | 7 | Diarrhea/vomiting | No |
| 13 | Yes | 48 | F | 2 | 6,10 | Higher | Fulltime | 20 | 13 | Fever | No |
| 14 | Yes | 33 | F | 2 | 1,5 | Higher | Fulltime | 16 | 5 | Fever | No |
| 15 | No | 41 | F | 2 | Missing | Higher | Fulltime | 16 | Missing | Respiratory symptoms | Yes |
| 16 | No | 37 | F | 1 | 2 | Secondary | Fulltime | 14 | 8 | Fever | No |
| 17 | No | 38 | F | 4 | 1,7,14,18 | Lower | Fulltime | 12 | 6 | Fever | Yes |
| 18 | No | 28 | F | 1 | 2 | Higher | Studying | 13 | 5 | Diarrhea/vomiting | No |
| 19 | No | 32 | F | 2 | 0,3 | Secondary | Maternity leave | 9 | 9 | Eye infection | No |
| 20 | No | 29 | F | 1 | 1 | Secondary | Fulltime | 13 | 6 | Fever | No |
| 21 | No | 42 | F | 2 | 2,2 | Higher | Fulltime | 16 | 6 | Fever | No |
| 22 | No | 30 | F | 1 | 2 | Secondary | Fulltime | 12 | 9 | Other | Yes |
| 23 | No | 37 | M | 2 | 9,11 | Higher | Fulltime | 16 | 7 | Other | Yes |
| 24 | No | 37 | M | 1 | 1 | Secondary | Fulltime | 12 | 4 | Rash | No |
| 25 | No | 43 | F | 1 | 8 | Secondary | Fulltime | 22 | 6 | Injury | Yes |
| 26 | No | 52 | M | 5 | 1,7,15,16,20 | Lower | Fulltime | 12 | 6 | Respiratory symptoms | No |
| 27 | No | 32 | F | 2 | 0,2 | Higher | Maternity leave | 17 | 5 | Fever | No |
| 28 | No | 47 | F | 2 | 11,13 | Higher | Fulltime | 15 | 6 | Injury | Yes |
| 29 | No | 36 | F | 3 | 3,3,7 | Higher | Fulltime | 17 | 5 | Fever | No |
| 30 | No | 42 | F | 1 | 1 | Higher | Fulltime | 15 | 5 | Fever | Yes |
| 31 | No | 30 | F | 1 | 2 | Higher | Fulltime | 17 | 5 | Fever | Yes |
| 32 | No | 44 | F | 2 | 4,5 | Higher | Fulltime | 23 | 3 | Fever | No |
| 33 | No | 43 | M | 2 | 4,11 | Secondary | Fulltime | 14 | 3 | Respiratory symptoms | Yes |
| 34 | No | 40 | M | 1 | 3 | Higher | Fulltime | 17 | 4 | Swollen toungue | No |
| 35 | No | 53 | M | 1 | 2 | Secondary | Fulltime | 17 | 8 | Injury | No |
| 36 | No | 37 | M | 2 | 4 | Secondary | Fulltime | 24 | 6 | Fever | No |
| 37 | No | 46 | M | 1 | 4 | Secondary | Unemployed | 28 | 7 | Fever | No |
| 38 | No | 56 | M | 1 | 3 | Higher | Fulltime | 27 | 7 | Respiratory symptoms | Yes |
| 39 | No | 42 | F | 1 | 6 | Secondary | Fulltime | 16 | 7 | Injury | No |

**Text message for inclusion**

Dear Parent,

You have recently been in contact with MH1813 regarding your ill child. We are a team of doctors and nurses in the Capital Region dedicated to improving health services for parents dealing with a sick child. Therefore, we would like to know if a pediatrician can call you to ask a few brief questions. This will take approximately 10-15 minutes. Please click on the link for more information and to inform us if we may contact you:

https://redcap.link/BB

Thank you for your time.

**Consent Declaration and Reflection Prompts:**

Your answers will help us research how to better create information for parents when their children are ill. Do you consent to a pediatrician contacting you for a short interview (10-15 min)? (Yes/No)

Which phone number would you prefer for contact:

It would greatly assist us if you could consider completing the following sentences before our call:

(You are also welcome to add comments in the field)

1) When I think back to the situation in which I contacted MH1813, the first thing that comes to mind is ...

2) Reflecting on the situation where I contacted MH1813, the purpose of the call was….

3) When I received a voice message about informative videos, I thought...

4) I chose/chose not to receive videos because...

5) The possibility of receiving informational materials about acute illness in children when contacting 1813 is...

6) If I am uncertain about what to do when my child is sick, I usually...

If you have any questions, feel free to write in the field or reach out to: (e-mail address for LB)

Thank you for your response.

**Interview Guideline**

Introduction

Presentation of the researcher (Liv Borch-Johnsen, MD and Ph.D. student), the purpose of the interview, and a word of thanks to the participants:

*My name is Liv. I am a doctor at MH1813 and at Hvidovre Children's Department.*

*I have sent you an SMS that you have been kind enough to answer.*

*Is that alright with you now, or should I call a little later?*

*First of all, I would like to thank you for allowing us to contact you.*

*The purpose of the interview is to gain more knowledge about how we, as health professionals, can best prepare and present health information for parents with acutely ill children. This is also why we have reached out to you, as you recently contacted 1813.*

*It takes approximately 15 minutes. I will record the conversation, but the recording will be deleted as soon as I have noted down the answers. Is that alright with you?*

Personal questions:

*To start off, I would like to ask you a few brief questions about yourself. For the record, you should know that we do not store any personal information, such as your phone number, name, or email. I ask these questions because I want to ensure that I interview a diverse group of parents, including both genders, first-time parents, and those with multiple children, among others.*

*1) How old are you?*

*2) How many children do you have?*

*3) Could you please describe your education and current employment status (e.g., student, full-time employee, part-time employee, unemployed)?*

- *Now, I would like to ask you a little about the circumstances surrounding your call to MH1813. You called 1813 on (date of the call). • Do you remember what symptoms your child had?*
- *How did they present themselves?*
- *Is this something you have experienced previously?*
- *Had you taken any action before the call?*
- *What was the purpose of contacting 1813?*
- *Was there anything in particular that concerned you?*

The 14 parents who participated in the randomized clinical trial and viewed the informative video tutorials titled Tips by Pediatricians were asked the following questions:

- *During the call, you chose to receive the pediatrician's child tips. The call was then disconnected, and you received a link to eight videos. When you received the link, what did you decide to do? • Which movie did you watch first?*
- *Did you explore other videos? Why or why not?*
- *What did you do after watching the videos?*
- *Which pieces of advice could you apply to your situation?*
- *How did watching the videos change your perspective?*
- *Did you discuss the content of the videos with your partner? If so, what consequences did this conversation have?*
- *Was there anything particularly good about the videos? Was there anything you found less favorable?*
- *Did you encounter anything in the videos that you did not understand?*
- *Could you utilize the advice again if your child made a similar mistake?*
- *How might this affect future situations, e.g., regarding when you would contact MH1813?*
- *The videos describe symptoms in children with sudden illness and are categorized based on the most common symptoms. Did this categorization make sense to you?*
- *What were your thoughts about the call being disconnected?*
- *What does it mean to you not to have the option of talking to a nurse or a doctor?*
- *If you had the opportunity to view the videos beforehand, do you believe you would have chosen to contact 1813 in the first place? Why or why not?*
- *Where should the videos be accessible so that you could find them before the actual call?*
- *Did you call 1813 back after viewing the videos? Why or why not?*

The 25 parents who declined to participate in the RCT but received telephone triage by a nurse or doctor were asked the following questions:

- We would like to know more about what worries parents when their children are acutely ill and where they find answers to their questions: • What worries you when your child is sick?
- What do you usually do when your child is sick?
- If you want to know more, where do you typically go? Which websites do you usually visit and what do you search for?
- We have created a series of videos for parents of children with acute illnesses such as fever, vomiting, diarrhea, and more. Does this sound like something you would find helpful?
- When would these videos be relevant to you?
- Where should they be placed for easy access when your child is ill?

Both interviews concluded with the following questions:

- I have no more questions. Is there anything else you would like to share about your 1813 call?
- Is there anything you think I should have asked you?
- May I contact you again within 14 days with any follow-up questions?
- Thank you. If you have any questions later, you are welcome to write to the email address you found in the questionnaire.
